# High rebound mattress toppers facilitate core body temperature drop and enhance deep sleep in the initial phase of nocturnal sleep

**DOI:** 10.1371/journal.pone.0197521

**Published:** 2018-06-27

**Authors:** Shintaro Chiba, Tomoko Yagi, Motohiro Ozone, Mari Matsumura, Hirofumi Sekiguchi, Masashi Ganeko, Sunao Uchida, Seiji Nishino

**Affiliations:** 1 Department of Otorhinolaryngology, Jikei University School of Medicine, Tokyo, Japan; 2 Stanford Sleep and Circadian Neurobiology Laboratory, Stanford University School of Medicine, Palo Alto, California, United States of America; 3 Ota Memorial Sleep Center, Ota General Hospital, Kawasaki, Kanagawa, Japan; 4 Department of Psychiatry, Jikei University School of Medicine, Tokyo, Japan; 5 Faculty of Business and Information Sciences, Jobu University, Isesaki, Gunma, Japan; 6 Faculty of Sport Sciences, Waseda University, Tokorozawa, Saitama, Japan; Charité - Universitätsmedizin Berlin, GERMANY

## Abstract

Recently, several new materials for mattresses have been introduced. Although some of these, such as low rebound (pressure-absorbing/memory foam) and high rebound mattresses have fairly different characteristics, effects of these mattresses on sleep have never been scientifically evaluated. In the current study, we have evaluated effects of a high rebound mattress topper [HR] on sleep and its associated physiology, and the effects were compared to those of a low rebound mattress toppers (LR) in healthy young (n = 10) and old (n = 20) adult males with a randomized, single-blind, cross over design. We found that sleeping with HR compared to LR induced a larger decline in core body temperature (CBT) in the initial phase of nocturnal sleep both in young (minimum CBT: 36.05 vs 36.35°C) and old (minimum CBT: 36.47 vs. 36.55°C) subjects, and declines in the CBT were associated with increases in deep sleep/delta power (+27.8% in young and +24.7% in old subjects between 11:00–01:00). We also found significantly smaller muscle activities during roll over motions with HR (-53.0 to -66.1%, depending on the muscle) during a separate daytime testing. These results suggest that sleeping with HR in comparison to with LR, may facilitate restorative sleep at the initial phase of sleep.

## Introduction

Sleep is fragile and can easily be affected intrinsically and by the environment. While an unpleasant sleep environment negatively affects the quality of sleep, increasing the sleep quality by choosing the optimal sleep environment has not garnered enough attention. This includes bedding; very little studies have scientifically evaluated the effect of bedding on sleep and physiological changes during sleep (see, [1, 2]). In addition to traditional innerspring mattresses, several types of mattresses with new materials have been introduced recently. Although some of these mattress types such as low rebound, or pressure-absorbing/memory foam, and high rebound, a polyethylene fiber-resin based mattress that has a firm, supportive feel, have fairly different characteristics, effects of these mattresses on sleep have never been compared scientifically.

In the current study, we have evaluated effects of a high rebound mattress topper [HR] on sleep and its associated physiology, and the effects were compared to those of a low rebound mattress topper (LR) with a randomized single-blind cross over design. The evaluations of mattress toppers included an overnight standard sleep polysomnograph (PSG) with the following parameters: continuous electroencephalograms (EEG), electrooculograms (EOG), electromyograms (EMG), heart rate, and core body (rectal) temperature. The following morning, objective (psychomotor vigilance test [PVT] [3, 4]) and subjective evaluations (questionnaires and visual analogue scales [VAS] [5] of sleep status and performance) were administered. Urinary growth hormone (GH) levels were measured. Two sets of subjects were included. Study I included ten healthy young adult male subjects, and study II included twenty healthy old adult male subjects. In a separate study, we also evaluated muscle activities that are needed to generate a roll over motion on HR and LR during the daytime in eight healthy young males.

## Materials and methods

### Materials

For HR, polyethylene fiber-resin-based mattress toppers (airweave® toppers, airweave inc., Tokyo, Japan) and for LR, urethane-based memory foam mattress toppers (Topper Deluxe 3.5, TEMPUR-SEALY Japan Ltd., Kobe, Japan) were used. Both toppers were commercially available in Japan. The topper sizes were approximately the same (HR: 195x100x3.5 cm, LR: 195x97x3.5 cm), and HR was 16% lighter than LR (HR: 6.7 kg, LR: 8.0kg). The toppers were placed on top of regular mattresses/beds equipped at the Ota Memorial Sleep Center, Kawasaki, Japan, where the PSG was carried out (S1 Fig).

### Subjects

Two subject groups, one consisting of ten healthy young adults (study I) and another of twenty health old adults (study II), were included for sleep evaluation. In a separate study, eight healthy young adult male subjects were evaluated for muscle activity during roll over motions during the daytime. The study was approved by the institutional review board (IRB) of Ota Memorial Sleep Center (protocol #11005, approved 5/27/2011 and protocol #11011, approved 11/9/2011), and all subjects provided informed consent. All subjects were blind to the information of the mattress toppers used.

a) Study I sleep evaluations

Ten healthy male subjects (26.7±7.7[SD] years old) with normal sleep habits were enrolled. Subjects between age 20 and 39 were recruited through a medical volunteer recruiting company (Souken, Tokyo, Japan), and 30 subjects without any sleep disorders, circadian rhythm disorders, or allergic rhinitis were initially selected. An acclimation PSG night was performed on each subject. Subjects with 3% Oxygen Desaturation Index (ODI) of > 5 per hour and respiratory disturbance index (RDI) of >5 per hour were excluded first, and 10 of the remaining subjects with the lowest Pittsburg Sleep Quality Index Score (PSQI) [6], were selected for enrollment. The mean height of the 10 subjects was 169.6±6.3m, weight, 61.9±5.2kg, BMI, 21.2±1.7, PSQI, 3.0±0.7 (range 0–6), 3% ODI, 2.3±1.1, and RDI, 1.8±1.2.

b) Study II sleep evaluations

Twenty healthy male subjects (61.2±3.2 years old) with normal sleep habits were enrolled. Subjects between age 55 and 65 were recruited through a medical volunteer recruiting company (Souken, Tokyo, Japan), and 30 subjects without any sleep disorders, circadian rhythm disorders, or allergic rhinitis were initially selected. An acclimation PSG night was performed in subjects. Subjects with 3% ODI of > 15 per hour and RDI of >15 per hour were excluded first, and 20 of the remaining subjects with the lowest PSQI were enrolled. The mean height of the 20 subjects was 169.2±5.8m, weight, 61.6±5.4kg, BMI, 21.6±2.0, PSQI, 5.8±3.5 (range 0–13), 3% ODI, 7.5±4.1, and RDI, 6.8±3.8.

Study I was conducted in January and February of 2011, and study II was conducted in September and October of 2012. The Ota Memorial Sleep Center is a sleep clinic equipped with 9 PSG recording rooms, and all rooms were air conditioned throughout the year. Room temperatures of the sleep recording rooms logged 25.15± 0.64°C at 21:00 and 23.55± 0.66°C at 09:00 in January/February (study I) and 25.70± 0.87°C at 21:00 and 25.10±0.72°C at 09:00 in September/October (study II).

c) EMG activity measures during roll over motion

In addition to evaluating position changes during the PSG (see below), EMG activity during roll over motions were evaluated on each mattress topper during the daytime without concurrent PSG recordings. Eight healthy males (20.8±0.5 years old) were enrolled. All subjects were students enrolled in Waseda University who volunteered, and they reported no orthopedic and neurological problems. This was also confirmed by a MD (one of the authors). The mean height of the 8 subjects was 172.3±11.1m, weight, 66.6±10.3kg, and BMI, 22.4±2.0.

### Study designs

a) PSG and evaluation of sleep related physiology

Sleep evaluations were carried out with a randomized, single-blind, cross over design. Subjects completed one night of PSG (23:00 to 7:00) per each topper for both toppers at the sleep laboratory of Ota Memorial Sleep Center, with 1–2 day(s) between each arm (S1 Fig).

A standard PSG consisting of EEGs from C3, C4, O1, O2, F3, and F4; EOG; submentalis EMG; chest electrocardiograms (ECG); and respiration curves derived by an oronasal thermistor and flowmeter (for an acclimation PSG night) was recorded using the Sandman system (Tyco Healthcare Japan, Inc. Tokyo, Japan) throughout the night. Sleep stages were scored every 30 seconds using the criteria described by Rechtschaffen and Kales [7]. Sleep onset was defined as the first epoch in which one of the sleep stages (Stage 1, 2, 3, 4, REM) were present after lights out. Final wakeup time was defined as the awakening time before lights on.

In addition to the PSG, the number of position changes during sleep, autonomic nerve activity (by monitoring ECG heart rate variability), core body temperature monitoring, and nighttime urinary GH levels were assessed. The rectal temperature was measured using a 401J thermistor probe (Nikkiso-Therm, Tokyo, Japan), and the data was captured with a NY Logger (N542R, Nikkiso) with a sampling rate of 1Hz. To measure the urinary GH levels secreted during the night, urine was collected in the morning when subjects woke up. In cases where subjects urinated during the night, all samples from during the night and morning were pooled for analysis. Urinary GH levels were measured using chemiluminescence immunoassay and corrected for creatinine [8]. VAS of sleep status (VAS-S), VAS performance (VAS-P), and the Stanford sleepiness scale (SSS) [9] were administered the following morning between 7:00 and 8:00 for subjective sleep evaluations (S1 Fig). Objective performance was evaluated with a psychomotor vigilance test (PVT) using a PVT task monitor (PVT192, CWE, Inc. Ardmore, PA, USA) [3, 4] (S1 Fig). Mean reaction time (RT), median RT, minimum RT, maximum RT, and lapses (RT > 500ms) were analyzed.

Sympathetic nerve activity was estimated from the heart rate variability of the ECG data. For heart rate variability measurements, high-frequency (HF; 0.15–0.4 Hz) and low-frequency (LF; 0.04–0.15 Hz) components of heart rate variability were obtained using MemCalc/win (Suwa Trust, Tokyo, Japan), a time series analysis technique software that combines the non-linear least square method with the maximum entropy method [10, 11]. The LF/HF value was calculated for every minute and averaged across each 2-hour time bin or the total 8 hours.

EEG delta wave power was obtained with MemCalc/SyUn (Suwa Trust, Tokyo, Japan), a version of MemCalc specialized for EEG data analysis. The EEG data (C3 and C4, sampling rate: 256Hz, 0.3Hz low cut, 35Hz high cut filters captured with the Sandman system) were processed consecutively, and the spectral power(μV2) in delta wave range (1–4 Hz) was computed for every 30 sec and were summed for each 2-hour time bin for each subject. The mean delta power of 10 (study I) and 20 (study II) subjects for each 2-hour time bin was compared between HR and LR. The results of power analysis of the C3 and C4 leads were equivalent, and the results of C3 are presented in this manuscript.

To evaluate the number of times subjects rolled over during sleep, a body position sensor (Pro-Tech, Woodinville, WA), which can distinguish five body positions (supine, prone, left lateral decubitus, right lateral decubitus, and sitting) was attached to the subjects [12]. The sleep position was recorded onto a polygraph in a coded format every 15 seconds, and it was monitored in real time by polysomnographic technologists that were attending the study. The number of times subjects changed body positions during sleep was calculated, and the time elapsed between the position change and subjects falling back to sleep was measured for each roll over episode. Changes in body position for less than 30 seconds were not considered for analysis.

b) EMG activity measures during rolling over

Since continuous EMG recording of multiple muscles during sleep may disturb sleep, we measured the EMG activity when subjects rolled over on HR and LR during the daytime without sleep recordings[13].

Muscle activity of the trapezius muscle (right and left) and the abdominal oblique external muscle (right and left) were measured. Subjects were instructed to lay supine on the toppers and then roll leftwards until they reached a left lateral recumbent position. The subjects repeated this motion on two types of mattress toppers (HR and LR rebound) and on a blanket on the floor for 3 times for each condition. To reduce the use of limb strength on the turning over movements, subjects raised their hands and rolled over only with their upper body without moving their feet.

Two surface disposable Ag/AgCl electrodes (F-150S, Nihon Kohden, Tokyo, Japan) were placed 2 cm apart on the external oblique muscle (*Musculus obliquus externus abdomens*). Both electrodes were placed around the center of a line drawn between the inferior borders of the 10th rib and pubis and on the muscle belly under the 10th rib. For the trapezius muscle (*musculus trapezius)*, electrodes were placed 2cm apart on the upper fibers of the trapizius, approximately 2 cm to the lateral inferior direction of the *Vertebra prominens* (C7). EMG signals were amplified with AM-601G amplifier (Nihon Kohden) with 15Hz low cut and 3KZ high cut filters. The data were analyzed using Spike 2 (CED, Cambridge, England), a data analyzing software. EMG activity data during the roll over motion was put through the “Rectify” and “Smooth” functions, and duration (time), mean amplitude (mV), integrated EMG (mV x sec), maximum amplitude (mV) were calculated. For this study, we defined EMG activity pertaining to a single rollover movement as follows: (1) The beginning and end of the single roll over movement is defined as the beginning and end of the EMG activity that includes the max amplitude, (2) the beginning of roll over motion was defined as when the amplitude exceeded that of standard value of posterior position (0.0–0.5 sec). (standard value = mean amplitude+3SD), and (3) the end of the roll over motion was defined as when the amplitude became smaller than the standard value of when subjects were in the left lateral recumbent position (6.0–6.5 sec). When EMG activity greater than the standard value was seen multiple times within 20ms, these waves were regarded as a continuous wave.

For the analysis of sleep and sleep related physiology, the significance of difference in effects (HR and LR) was evaluated with the paired-t test, and the significance of difference in effects (young and old subject groups) was evaluated with the Student’s-t test. For the time sequence data (core body temperature, FFT, LF/HF and slow wave sleep amounts), the significance of the effects (topper type, time, topper type x time) was analyzed by applying the repeated measures ANOVA with a grouping factor (topper type). The core body temperature data were analyzed and displayed in 20-minute time bins, while FFT, LF/HF, and slow wave sleep data were analyzed and displayed in 2-hour time bins.

For the analysis of muscle activity during roll over motions, we calculated the average of the following for each subject for each experimental condition: duration (time), mean amplitude, integral, maximum amplitude. Mean integral for rolling on HR and LR were compared and presented. Student’s-t test was applied.

Systat (Systat Software Inc, San Jose, CA, USA) was used for data analysis and statistical significance was set at p<0.05.

## Results

**Fig 1:**
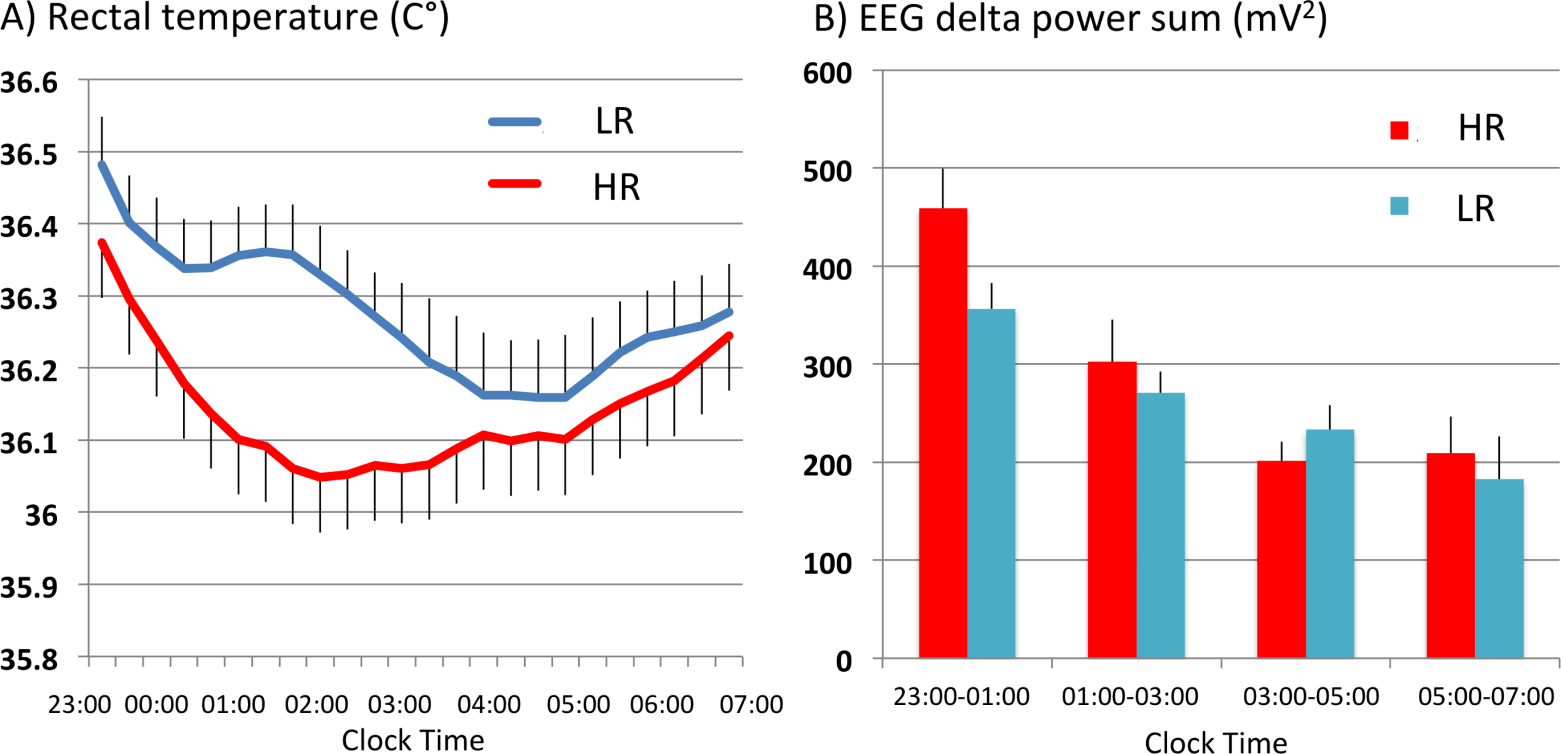
(A) Changes in core body temperature during sleep with HR and LR in young adult males (study I). Larger and longer lasting decrease in core body temperature (CBT) was seen in the initial half of the sleep period with HR (p<0.05 by topper type, p<0.01 by time, p<0.01 by topper type x time, repeated measures ANOVA with a grouping factor). (B) EEG delta power changes across the night (study I). Larger delta-power was observed with HR between 23:00–04:00 (p<0.01 by topper type, p = 0.22 by time, p<0.05 by topper type x time, repeated measures ANOVA with grouping factor).

Subjects for study I (10 young adult males) fell asleep more rapidly with HR (7.15±2.12 min) than with LR (9.10±2.61 min), but no statistical difference was observed (Table 1). Sleep and sleep-related parameters for the overall 8 hrs of the PSG showed little differences between HR and LR. REM latency tended to be shorter with HR (Table 1). Interestingly, the core body temperature rapidly and continuously decreased with HR until reaching the minimum (36.05°C) around 02:00. Decline of the temperature with LR was more modest and remained 0.1–0.3°C higher than that with HR between 00:00 to 03:00 and reached the minimum (36.15°C) at 04:00–05:00 am Topper (p<0.01) and topper type x time interaction (p< 0.01) were significant (Fig 1A). Since significant difference in core body temperature was observed in the first half of the recording time, we analyzed the PSG data for each 2-hour time bin, from 23:00 to 01:00, 01:00 to 03:00, 03:00 to 05:00, and 05:00 to 07:00. For stage 4 sleep, statistically significant topper type x time interactions (p<0.05) was seen, and stage 4 sleep was longer with HR (9.4 min [23:00–01:00] and 8.5 min [01:00–03:00]) than with LR (1.9 min [23:00–01:00] and 3.9 min [01:00–03:00]) between 23:00 and 03:00 (Table 2). Concurrently, delta EEG power between 23:00 and 03:00 was higher with HR than with LR, and a significant difference in EEG delta power change over time was observed between the two toppers (p< 0.01; topper, p<0.05; topper type x time) (Fig 1B). In addition, sympathetic nerve activity was lower with HR between 23:00 and 01:00, and sympathetic nerve activity (LF/HF ratio of heart rate intervals) changes over time significantly differed between the two toppers (p<0.05, topper type x time) (Table 2).

**Table 1:** Comparisons of sleep and sleep-related parameters between HR and LR mattress topper use (study I).

	**SPT**	**TST**	**SL**	**REM latency**	**SE1 (TST/SPT)**	**%stage1 (/TST)**			**%stage2 (/TST)**		**%stage34 (/TST)**	**%stageREM (/TST)**
HR	470.85±2.50	428.60±17.27	7.05±2.12	75.80±14.43	91.01±3.61	8.29±0.50			56.63±1.81		11.32±1.15	23.81±2.08
LR	470.90±2.61	443.00±7.74	9.10±2.61	93.65±17.73	94.11±1.79	8.83±0.76			52.84±2.41		12.65±1.23	25.68±2.60
p-value	0.84	0.64	0.46	0.07	0.81	0.64			0.16		0.18	0.40
	**SE2 (TST/TIB)**	**%stageWAKE (/SPT)**	**%stage1 (/SPT)**	**%stage2 (SPT)**	**%stage34 (/SPT)**	**%stageREM (/SPT)**			**arousal index**		**Roll over during sleep**	**wake (s) after rolling over**
HR	89.29±3.60	9.45±3.70	7.53±0.54	51.06±1.43	10.24±1.07	22.23±2.39			4.65±0.64		3.00±0.93	30.50±7.27
LR	92.28±1.62	5.88±1.79	8.24±0.63	49.70±2.28	11.81±1.03	24.38±2.57			4.33±0.38		3.60±1.12	39.10±13.31
p-value	0.60	0.14	0.31	0.94	0.12	0.43			0.70		0.26	0.77
	**VASS**	**SSS**	**VASP**	**SSS2**	**VASP2**	**GH**			**LF/HF**			
HR	7.27±0.60	2.60±0.22	7.59±0.42	1.20±0.13	8.97±0.35	7.73±1.19			5.09±0.16			
LR	5.93±0.75	2.60±0.16	6.51±0.66	1.30±0.15	8.35±0.63	7.25±1.03			4.69±0.30			
p-value	0.07	0.93	0.05	0.34	0.11	0.58			0.26			
	**PVT Mean RT**	**PVT Median RT**	**PVT Minimum RT**	**PVT Maximum RT**	**Lapses (RT > 500ms)**							
HR	235.2±6.4	221.6±6.0	161.8±5.0	512.7±43.0	0.60±0.22							
LR	236.8±8.6	225.2±8.0	163.5±5.4	462.6±37.8	0.30±0.21							
p-value	0.93	0.71	0.90	0.38	0.08							

SPT, total sleep period (sleep period time); TST, total sleep time; SL, sleep latency; SE1, sleep efficiency; SE2, sleep efficiency; VASS, visual analogue scale sleep; VASP, visual analogue scale performance; SSS, Stanford sleepiness scale; GH, growth hormone; LF, heart rate variability low-frequency; HF, heart rate variability high-frequency; RT, reaction time. Statistical significance was evaluated by paired t-test.

**Table 2:** Amounts of slow wave sleep and sympathetic nerve activity (LF/HF values) in each 2-hour period (study I).

Parameters	Topper	Time bins					Difference (p value)	
		11:00–01:00	01:00–03:00	03:00–05:00	05:00–07:00	Topper	Time	Topper type x Time
Stage III (min)	HR	47.9±6.3	23.8±5.5	7.9±2.4	10.3±3.4	0.11	<0.01	0.67
	LR	44.5±0.7	27.0±5.5	1.9±0.9	2.9±2.9			
Stage IV (min)	HR	9.4±1.1	8.5±5.0	0.1±0.1	3.0±3.0	0.20	<0.01	<0.05
	LR	1.9±0.9	3.9±1.9	2.6±2.0	0.6±0.6			
LF/HF	HR	2.49±0.21	3.05±0.38	3.27±0.46	3.39±0.47	0.58	0.19	<0.05
	LR	3.72±0.26	3.17±0.45	2.89±0.29	3.42±0.38			

In young subjects, subjective sleep status and performance (VAS) of the morning after the PSG study tended to be better with HR than with LR, though not at significant levels (Table 1). No significant differences in SSS and PVT were noted, while the mean number of PVT lapses (RT>500 ms) had non-significant tendency to be better with LR than with HR (Table 1). No significant change in urinary GH secretion during the night was observed (Table 1).

In the current study, we also evaluated the effects of toppers on sleep and sleep-related physiology in the old adult subjects. It is known that sleep quality declines in the elderly, and the temperature changes during sleep are often dysregulated [14–16]. We also observed reduced sleep qualities of old adult subjects, including reductions in total sleep time, sleep efficiency, deep sleep (% stage 3&4), and REM sleep (S1 and S2 Tables). A tendency for reduced delta-power was observed in the old subjects between 11:00–03:00, the first half of sleep (p = 0.19 by age group, p<0.01 by time, p = 0.06 by by age group x time) (S2 Fig). Increases in % stage wake, arousal index, and stage 1 along with increase in subjective sleepiness (SSS2) in the morning were observed. Increase in the incidences of roll over during sleep was also noted in the old adult subjects. In the old subjects, all parameters from the PVT were significantly prolonged (S1 and S2 Tables). Core body temperature changes through the night were also distinct in the two age groups. In contrast to the initial decline of the temperature in the young subjects, core body temperature in old subjects was mostly unchanged until the second half of the nocturnal sleep (p<0.01 by age group, p<0.01 by time, p<0.01 by age group x time) (S2 Fig).

**Fig 2:**
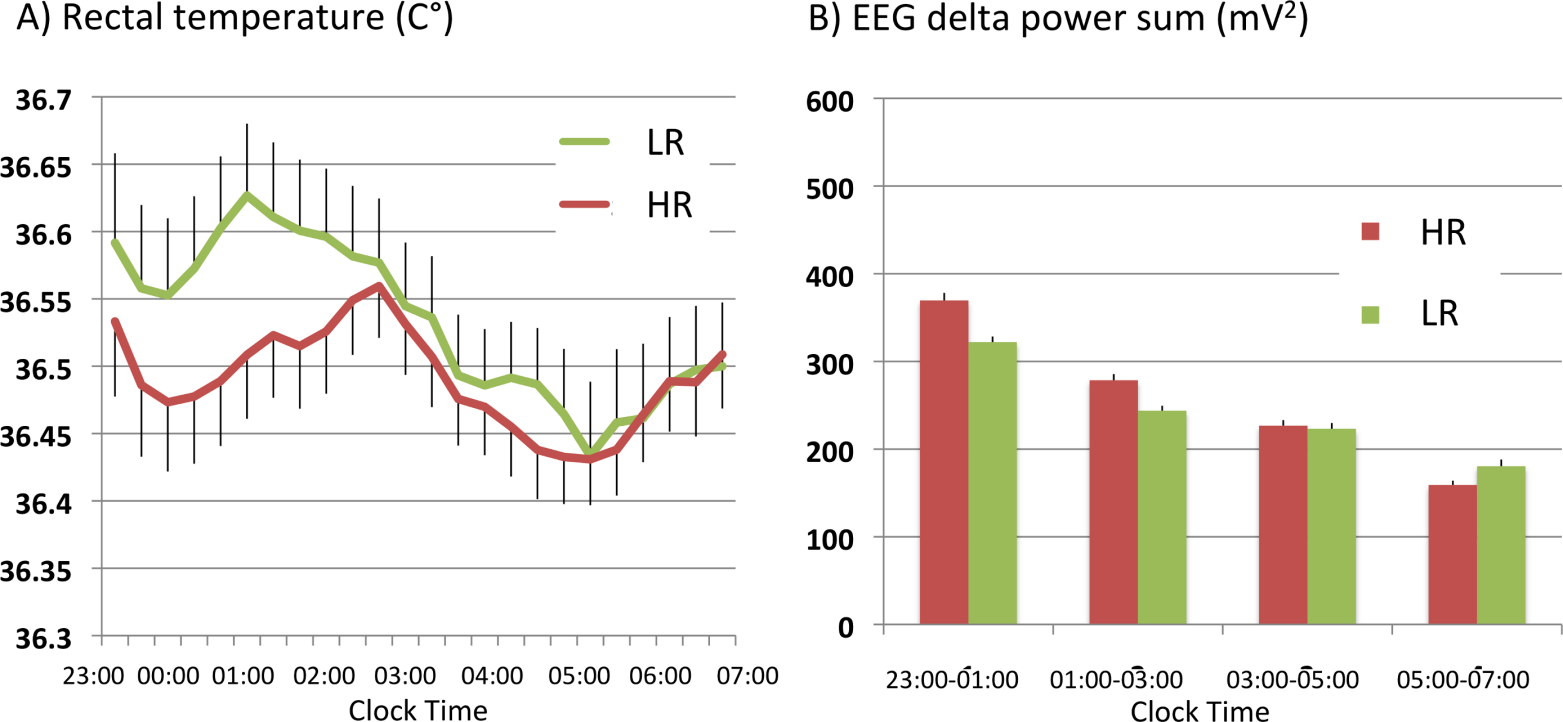
(A) Changes in core body temperature during sleep with HR and LR in old adult males (study II). As observed in younger subjects (Fig 2), larger decline in core body temperature (CBT) was observed with HR between 23:00–03:00 in old subjects (p<0.01 by topper type, p<0.02 by time, p<0.01 by topper type x time, repeated measures ANOVA with grouping factor). (B) EEG delta power changes across the night (study II). Larger delta-power was observed with HR between 23:00–03:00 (p<0.01 by topper type, p = 0.23 by time, p<0.05 by topper type x time, repeated measures ANOVA with grouping factor).

In the old subjects, a larger decline in the core body temperature (CBT) was also observed with HR than with LR (study II: 20 old adult males) during 23:00–03:00, while the decline of the temperature with both toppers was smaller and shorter-lasting (the minimum: 36.47°C between 00:00 to 01:00) compared to those in young subjects (the minimum: 36.05 around 02:00). CBT remained 0.05–0.1C° higher with LR than that with HR between 23:00 and 02:00 (Fig 2A), and statistical significances (p<0.01 by topper type, p<0.02 by time, p<0.01 by topper type x time) were observed. As stated above, the old subjects had small amounts of slow wave sleep (SWS) during this period (HR: 14.3 min (stage 3), 1.1 min (stage 4), LR: 14.7 min (stage 3), 0.5 min (stage 4)), but there was no difference between HR and LR use (S1 Table). However, delta EEG power between 23:00 and 1:00 was 17% higher with HR compared to LR, and a significant difference in EEG delta power change over time was observed between the two toppers ((p<0.01 by topper type, p<0.05 by topper type x time) (Fig 2B), similarly to the younger subjects (study I). We did not observe any significant differences between HR and LR in other PSG, VAS, and PVT parameters (Table 3). There was no significant change in nighttime urinary GH secretion (Table 3). In contrast to young subjects, no significant decline in LF/HF values were seen in the initial phase of sleep in the old subjects (S1 Table).

**Table 3:** Comparisons of sleep and sleep-related parameters between HR and LR mattress topper use (study II).

	**SPT**	**TST**	**SL**	**REM latency**	**SE 1 (TST/SPT)**	**%stage1 (/TST)**	**%stage2 (/TST)**	**%stage34 (/TST)**	**%stageREM (/TST)**
HR	459.58±7.14	382.30±13.32	6.73±2.12	83.53±11.83	82.99±2.36	21.29±1.97	52.75±1.92	4.07±0.93	21.92±2.00
LR	462.50±8.26	389.64±15.90	7.05±2.38	95.28±12.10	83.56±2.75	21.78±2.756	53.32±1.58	3.68±0.88	21.22±2.25
p-value	0.79	0.73	0.92	0.49	0.88	0.88	0.82	0.76	0.74
	**SE 2 (TST/TIB)**	**%stageWAKE (/SPT)**	**%stage1 (/SPT)**	**%stage2 (SPT)**	**%stage34 (/SPT)**	**%stageREM (/SPT)**	**arousal index**	**Roll over during sleep**	**wake (s) after rolling over**
HR	79.46±2.78	20.50±3.31	17.30±1.50	49.04±5.73	3.27±0.71	18.38±1.38	10.94±1.38	6.80±1.31	52.48±6.72
LR	81.17±3.31	19.44±4.42	17.58±1.53	44.60±2.08	3.06±0.71	18.33±1.66	10.44±1.44	6.35±1.66	50.60±11.72
p-value	0.72	0.85	0.9	0.47	0.84	0.84	0.8	0.83	0.99
	**VASS**	**SSS**	**VASP**	**SSS2**	**VASP2**	**GH**	**LF/HF**		
HR	6.72±0.51	2.45±0.20	6.70±0.48	1.70±0.15	8.17±0.32	5.92±0.78	4.76±0.16		
LR	7.02±0.52	2.50±0.20	6.84±0.361	1.75±0.14	8.05±0.30	7.48±1.12	4.79±0.13		
p-value	0.68	0.86	0.82	0.81	0.79	0.25	0.33		
	**PVT Mean RT**	**PVT Median RT**	**PVT Minimum RT**	**PVT Maximum RT**	**Lapses (RT > 500ms)**				
HR	256.9±4.2	239.3±4.4	180.5±2.9	659.4±48.9	1.90±0.38				
LR	257.4±4.3	238.5±3.9	184.6 ±2.9	691.6±67.2	1.85±0.41				
p-value	0.94	0.89	0.27	0.70	0.93				

SPT, total sleep period (sleep period time); TST, total sleep time; SL, sleep latency; SE1, sleep efficiency, SE2, sleep efficiency; VASS, visual analogue scale sleep; VASP, visual analogue scale performance; SSS, Stanford sleepiness scale; GH, growth hormone; LF, heart rate variability low-frequency; HF, heart rate variability high-frequency; RT, reaction time. Statistical significance was evaluated by paired t-test.

The old subjects also entered sleep rapidly with both HR (6.7±3.0 min) and LR (7.1±3.4 min), and no difference between HR and LR was observed (Table 3).

**Fig 3:**
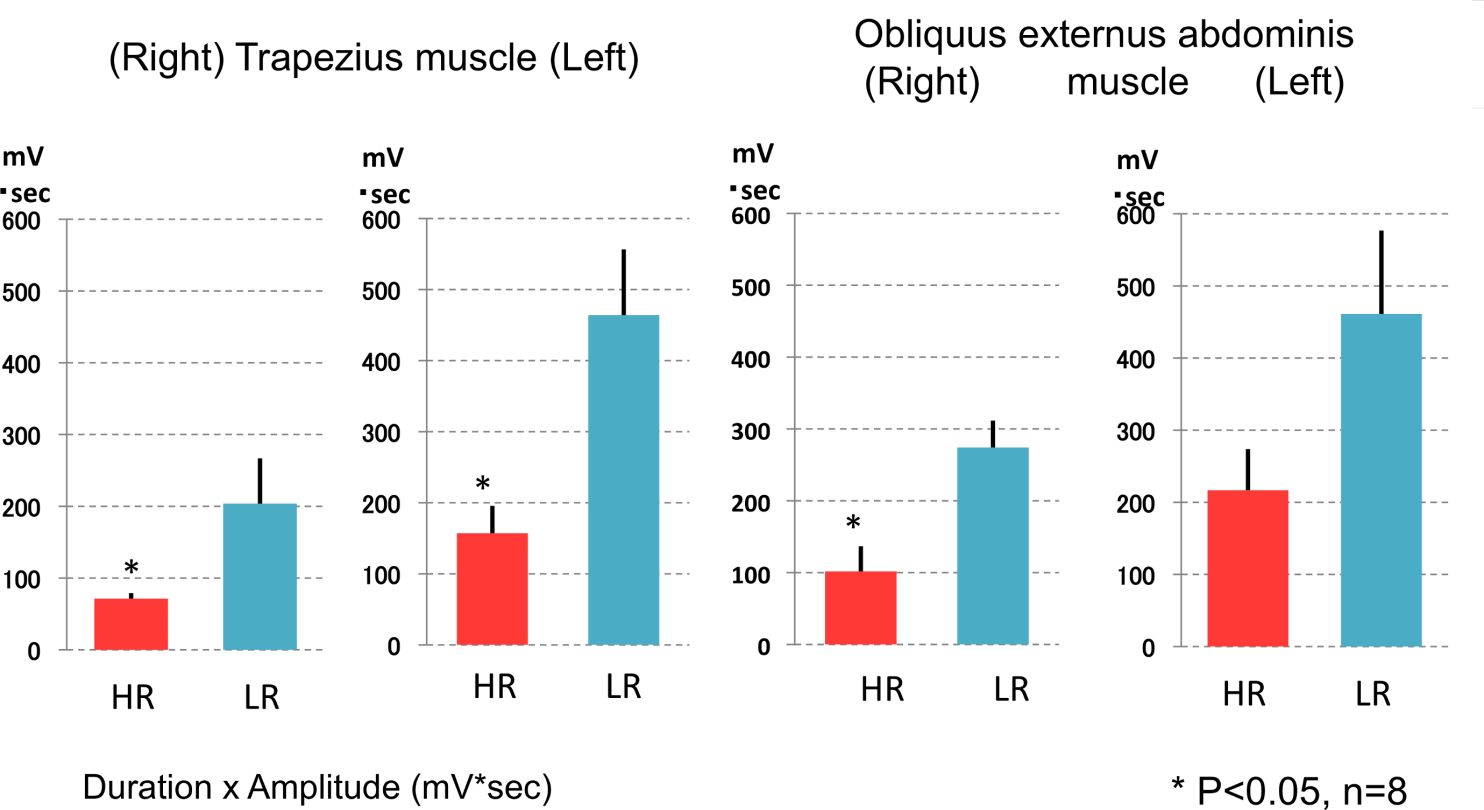
Muscle activity needed to generate a rolling over motion on HR and LR mattress (Roll over evaluations). Significantly smaller muscle activity (i.e., integrated EMG) was needed to roll over on HR than on LR mattress in 3 out of 4 muscles measured (*p<0.05 by Student’s t-test).

There were no significant differences in the numbers of position changes and in the mean time elapsed between the position change and falling back to sleep between the two topper types in both young and old subjects (Table 1 and Table 3). In a separate evaluation during the daytime with young subjects, we observed that much smaller muscle activity (i.e., integrated EMG) was needed in order to roll over on HR than on LR mattress toppers (Fig 3). Significant differences (-53.0 to -66.1%, compared to LR) were observed in the activities of 3 out of 4 muscles measured, and difference according to mattress type was observed in both the duration and mean amplitude of EMG, with more profound effects observed in the duration (data not shown).

## Discussions

The most significant difference we found between HR and LR was core body temperature changes in the initial phase of the sleep. In young subjects, the core body temperature rapidly and continuously decreased with HR until reaching the minimum (36.05°C) around 02:00. Temperature decline with LR was more modest; CBT with LR remained 0.3°C higher than that with HR between 01:00 to 02:00 and reached the minimum (36.15°C) in the early morning from 04:00 to 05:00 am. In the old subjects, temperature changes followed a similar pattern but were attenuated (0.1°C higher with LR at 01:00). Between 11:00 and 03:00, body temperature was lower with HR compared to LR, and HR increased stage 4 sleep (for study I) and/or EEG delta power (study I and II). Therefore, significant declines in the core body temperature with HR were observed regardless of the ages and in the different temperature profiles (S2 Fig).

In contrast to the core body temperature changes, effects on sleep stages were subtle (Tables 1 and 3). Significance was observed only in the increase in stage 4 in young subjects with HR in the first half of the sleep, which was associated with decrease in sympathetic nerve activity. We however observed increase in EEG delta power in the first half of sleep in both young and old subjects with HR suggesting that sleeping with HR induced EEG-defined deeper sleep at the first half of sleep regardless of age.

A series of experimental evidences suggested the functional importance of the first NREM sleep cycle for sleep [17, 18]. For example, the NREM sleep propensity, which is reflected in increased EEG delta power, is highest at the sleep onset, and the deepest NREM Sleep occurred at the first NREM cycle [18, 19]. NREM sleep propensity gradually declines toward the morning until subjects wake up [18, 19]. During the first NREM sleep cycle, GH, which stimulates growth, cell reproduction, cell regeneration, and metabolism, is secreted [20]. This GH secretion is mostly sleep-state dependent, and disruptions of the first cycle NREM sleep interferes with normal GH secretion [20]. Decrease in sympathetic nerve activity was also associated with deep sleep, and the decline of sympathetic nerve activity is largest at the first NREM sleep cycle [21]. It is also known that disruption of the first NREM sleep cycle disturbs the whole-night sleep wake cycle (see, [22]), and the first NREM sleep cycle is often bypassed in patients with psychiatric disorders (see, [17]). Therefore, even if sleep effects with HR are subtle, increases in EEG delta power in the initial half of the sleep likely merits the subjects.

Studies have also shown that temperature regulation before sleep and the initial phase of sleep is very import for sleep induction and sleep maintenance [23, 24]. Temperature is primarily regulated through two factors, namely heat production and heat loss [23]. The core body temperature is high during the daytime and low at night [24]. In contrast, distal skin temperature is low during the daytime and high at night [24]. The core body temperature is about 2°C higher than the distal skin temperature [24]. Proximal skin temperature behaves similarly to core body temperature, but is lower than core body temperature [24].

Previous studies have shown that before and around sleep onset, heat loss from the distal skin occurs, and this results in a decrease in core body temperature which facilitates sleep onset and induction of deep sleep [25]. Krauchi et al. had experimentally demonstrated that when DPG (distal-proximal skin temperature gradient) becomes smaller, people tend to fall in sleep [25]. Since the HR topper is constructed of polyethylene resin fibers that are interwoven to create the 3 dimensional structure of the topper, thus allowing airflow (see S1 Fig), we believe effective heat exchange of the body may have occurred when sleeping on HR. This may have induced the large decline in core body temperature we observed and facilitated occurrences of deep sleep at sleep onset during the first phase of sleep. In order to test the hypotheses that HR allows effective heat los thus leading to sleep onset facilitation and induction of deep sleep, it is important to measure DPG changes together with the core body temperature measures during PSG, and further experiments are warranted.

Another important characteristic we observed for HR is that muscle activity required for the rolling over motion is significantly smaller on HR compared to LR in a daytime experiment. During nocturnal sleep monitoring, we did not observe differences in number of times subjects rolled over between HR and LR in both study I and II. We also monitored mean elapsed time to sleep after position changes with HR and LR, but there was no difference. However, given that smaller muscle activity was required for each roll over event, this characteristic also likely aids the restorative function of sleep. In accordance with this interpretation, subjective performance (VASP) was significantly better and subjective sleep status (VASS) had a non-significant tendency to be better in the morning following the night of sleeping on HR. The HR and LR toppers we used were 3.5 cm thick, and surprisingly this thin layer topper (on top of the same mattresses) was enough to produce significant differences in temperature changes, roll over efforts, and quality of sleep.

Another aim for including the old adult subjects was to examine if HR aids subjects who have difficulty falling sleep. It has been postulated that dysregulation of body temperature at bedtime, especially in old subjects, may interfere with the sleep occurrence or quality [14, 15]. However, the old subjects we evaluated fell asleep with both HR and LR as quickly as the young subjects, and we did not detect a difference in sleep latency between HR and LR. Therefore, we need to include subjects with longer sleep latency (i.e., with difficulty falling asleep) to evaluate effects on sleep latency.

In conclusion, we found that sleeping with HR induced a large decline in core body temperature in the initial phase of nocturnal sleep both in young and old subjects, and declines in the core temperature were associated with increase in deep sleep/delta power after sleep onset. The sleep improvement we observed with HR was relatively small, but this effect may still contribute significantly for the restorative roles of sleep, as experimental evidences have suggested that the first NREM sleep cycle is of importance for functions of sleep. The smaller muscle activity required to roll over in a daytime experiment is another feature of HR that likely contributes to the restoration during sleep.

The limitations of the study should also be discussed. Firstly, the sample sizes of young (n = 10) and old (n = 20) subjects were small and not equal; larger numbers are likely required to detect more significant sleep effects. Secondly, the high room temperature (25.0–26.0°C at light off) during the sleep recordings is also to be noted. This is due to the temperature setting protocol of the sleep clinic the experiment took place in. Further evaluation is required if the results are reproducible at a regular, lower room temperature. Thirdly, we performed muscle activity measures during roll over motions in a separate daytime experiment to avoid sleep disruptions from involved EMG recordings during PSGs. The muscle activity during roll over motions during different sleep stages should also be evaluated.

Regardless of these limitations, our results suggest the possibility that types of bedding significantly affect sleep and its associated physiology, and bedding material/structures selection would likely improve sleep quality, therefore, further research is warranted.

## Supporting information

S1 Fig Experimental design and schedule and bed materials used. Effects of a high rebound mattress topper (HR) on sleep and its associated physiology were compared to those of a low rebound mattress topper (LR) with a randomized single-blind cross over design. Bottom figures: HR (left two figs) and LR (light fig) mattress toppers used in the study. nPSG; nocturnal polysomnography, PVT; psychomotor vigilance test, VAS; Visual analogue scale.
(JPG)

S2 Fig **(A) Changes in core body temperature during sleep in young (study I) and old (study II) subjects.** Compared to the young subjects, the decline of the temperature of the old subjects was not obvious and mostly unchanged until the second half of the nocturnal sleep (p<0.01 by age group, p<0.01 by time, p<0.01 by age group x time, repeated measures ANOVA with a grouping factor). (B) **EEG delta power changes across the night in young (study I) and old (study II) subjects.** Old subjects tended to have reduced delta-power between 11:00–03:00 (p = 0.19 by age group, p<0.01 by time, p = 0.06 by age group x time, repeated measures ANOVA with a grouping factor).
(JPG)

S1 Table Amounts of slow wave sleep and sympathetic nerve activity (LF/HF values) in each 2-hour period slept with HR and LR in old subjects (study II). (PDF)

S2 Table Comparisons of sleep and sleep-related parameters between young (study I) and old (study II) subjects. SPT, total sleep period (sleep period time); TST, total sleep time; SL, sleep latency; SE1, sleep efficiency, SE2, sleep efficiency; VASS, visual analogue scale sleep; VASP, visual analogue scale performance; SSS, Stanford sleepiness scale; GH, growth hormone; LF, heart rate variability low-frequency; HF, heart rate variability high-frequency; RT, reaction time. Statistical significance was evaluated by Student’s t-test.
(PDF)

S3 Table Amounts of slow wave sleep and sympathetic nerve activity (LF/HF values) in each 2-hour period slept in young (study I) and old (study II) subjects. (PDF)
