# Supplementary figures and images for: High rebound mattress toppers facilitate core body temperature drop and enhance deep sleep in the initial phase of nocturnal sleep
Source: PLoS One. 2018 Jun 27;13(6):e0197521. doi: 10.1371/journal.pone.0197521 (PMC6021054; doi:10.1371/journal.pone.0197521)

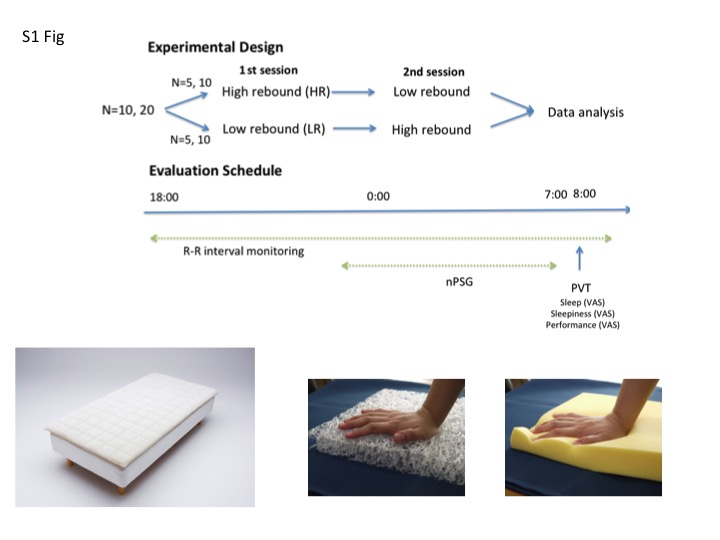

Supplement: S1 Fig — Effects of a high rebound mattress topper (HR) on sleep and its associated physiology were compared to those of a low rebound mattress topper (LR) with a randomized single-blind cross over design. Bottom figures: HR (left two figs) and LR (light fig) mattress toppers used in the study. nPSG; nocturnal polysomnography, PVT; psychomotor vigilance test, VAS; Visual analogue scale. (JPG) [file pone.0197521.s001.jpg]

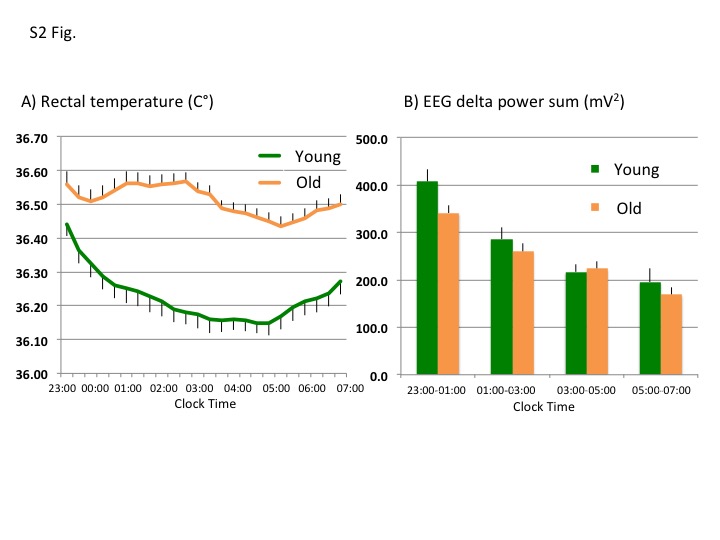

Supplement: S2 Fig — (A) Changes in core body temperature during sleep in young (study I) and old (study II) subjects. Compared to the young subjects, the decline of the temperature of the old subjects was not obvious and mostly unchanged until the second half of the nocturnal sleep (p<0.01 by age group, p<0.01 by time, p<0.01 by age group x time, repeated measures ANOVA with a grouping factor). (B) EEG delta power changes across the night in young (study I) and old (study II) subjects. Old subjects tended to have reduced delta-power between 11:00–03:00 (p = 0.19 by age group, p<0.01 by time, p = 0.06 by age group x time, repeated measures ANOVA with a grouping factor). (JPG) [file pone.0197521.s002.jpg]
