# Supplementary material for: High rebound mattress toppers facilitate core body temperature drop and enhance deep sleep in the initial phase of nocturnal sleep
Source: PLoS One. 2018 Jun 27;13(6):e0197521. doi: 10.1371/journal.pone.0197521 (PMC6021054; doi:10.1371/journal.pone.0197521)
Supplement: S1 Table — (PDF) [file pone.0197521.s003.pdf]

S1 Table. Amounts of slow wave sleep and sympathetic nerve activity (LF/HF values) in each 2-hour period slept with HR and LR in old subjects (study II).

Parameters	Topper	Time bins				Difference		
		11:00-01:00	01:00-03:00	03:00-05:00	05:00-07:00	Topper	Time	Topper type x Time
Stage III (min)	HR	14.3±3.3	6.7±2.6	5.4±2.0	3.5±2.1	0.78	<0.01	0.61
	LR	14.7±3.6	6.6±1.7	5.3±2.4	1.1±0.5			
Stage IV (min)	HR	1.1±0.9	0±0	0.9±0.6	0±0	0.59	0.35	0.69
	LR	0.5±0.4	0.3±0.3	0.4±0.3	0±0			
LF/HF	HR	4.55±0.79	4.66±0.70	4.52±0.90	4.43±0.51	0.57	0.97	0.87
	LR	4.68±0.87	4.28±0.61	3.85±0.55	3.66±0.38			
