# Supplementary material for: High rebound mattress toppers facilitate core body temperature drop and enhance deep sleep in the initial phase of nocturnal sleep
Source: PLoS One. 2018 Jun 27;13(6):e0197521. doi: 10.1371/journal.pone.0197521 (PMC6021054; doi:10.1371/journal.pone.0197521)
Supplement: S2 Table — SPT, total sleep period (sleep period time); TST, total sleep time; SL, sleep latency; SE1, sleep efficiency, SE2, sleep efficiency; VASS, visual analogue scale sleep; VASP, visual analogue scale performance; SSS, Stanford sleepiness scale; GH, growth hormone; LF, heart rate variability low-frequency; HF, heart rate variability high-frequency; RT, reaction time. Statistical significance was evaluated by Student’s t-test. (PDF) [file pone.0197521.s004.pdf]

S2 Table. Comparisons of sleep and sleep-related parameters between young (study I) and old (study II) subjects.

	SPT	TST	SL	REM latency	SE 1 (TST/SPT)	%stage1 (/TST)	%stage2 (/TST)	%stage34 (/TST)	%stageREM (/TST)
Yong	470.88±1.76	435.80±9,36	8.08±1.65	84,73±11.31	92.56±1.99	8.56±1.53	54.74±0.44	11.99±0.84	24.75±1.63
Old	461.04±5.39	385.97±10.26	6.73±1.50	89,40±8.40	8327±1.79	21.54±1.44	5303±1.23	3.87±0.63	21.57±1.05
p-value	0.21	<0.01	0.64	0.75	<0.01	<0.01	0.41	<0.01	0.10

	SE 2 (TST/TIB)	%stageWAKE (/SPT)	%stage1 (/SPT)	%stage2 (SPT)	%stage34 (/SPT)	%stageREM (/SPT)	arousal index	Roll over during sleep	wake (s) after rolling over
Young	90.79±1.95	7.67±2.04	7.89±0.41	50.38±1.32	11.03±0.75	23.31±1.72	4.49±0.37	3.30±0.71	34.47±7.07
Old	80.40±2.14	19.97±2.73	17.44±1.06	46.82±3.03	3.16±0.50	18.35±1.07	10.69±.99	6.58±1.05	51.48±7.13
p-value	<0.01	<0.01	<0.01	0.42	<0.01	<0.05	<0.01	<0.05	0.16

	VASS	SSS	VASP	SSS2	VASP2	GH	LF/HF	
Young	6.60±0.49	2.60±0.13	7.05±0.40	1.25±0.10	8.66±0.36	7.49±0.76	4.89±0.17	
Old	6.87±0.36	2.48±0.14	6.77±0.30	1.73±0.10	8.11±0.22	6.70±0.69	4.72±0.10	
p-value	0.66	0.57	0.58	<0.01	0.18	0.48	0.52	

	PVT Mean RT	PVT Median RT	PVT Minimum RT	PVT Maximum RT	Lapses (RT > 500ms)	
HR	236.0±5.2	223.4±4.9	162.7±3.6	487.6±28.4	0.45±0.15	
LR	257.2±3.0	238.9±2.9	182.3±2.0	675.5±41.1	1.88±0.28	
p-value	<0.01	<0.01	<0.01	<0.01	<0.01	

SPT, total sleep period (sleep period time); TST, total sleep time; SL, sleep latency; SE1, sleep efficiency, SE2, sleep efficiency; VASS, visual analogue scale sleep; VASP, visual analogue scale performance; SSS, Stanford sleepiness scale; GH, growth hormone; LF, heart rate variability low-frequency; HF, heart rate variability high-frequency; RT, reaction time. Statistical significance was evaluated by Student's t-test.
