# Supplementary material for: High rebound mattress toppers facilitate core body temperature drop and enhance deep sleep in the initial phase of nocturnal sleep
Source: PLoS One. 2018 Jun 27;13(6):e0197521. doi: 10.1371/journal.pone.0197521 (PMC6021054; doi:10.1371/journal.pone.0197521)
Supplement: S3 Table — (PDF) [file pone.0197521.s005.pdf]

S3 Table. Amounts of slow wave sleep and sympathetic nerve activity (LF/HF values) in each 2-hour period slept in young (study I) and old (study II) subjects.

.

Parameters	Topper	Time bins				Difference		
		11:00-01:00	01:00-3:00	03:00-05:00	05:00-07:00	Group	Time	Group x Time
Stage III (min)*	Young	46.2±4.6	25.4±3.8	4.9±1.4	6.6±2.3	<0.01	<0.01	<0.01
	Old	14.5±2.4	6.6±1.6	5.3±1.5	2.3±1.1			
Stage IV (min)	Young	6.6±3.0	10.6±2.4	1.4±1.0	1.8±1.5	<0.01	<0.01	<0.01
	Old	0.8±0.5	0.2±0.2	0.6±0.4	0±0			
LF/HF	Young	3.10±0.21	3.11±0.29	3.08±0.27	3.40±0.29	<0.05	0.82	0.45
	Old	4.61±0.58	4.47±0.46	4.18±0.52	4.04±0.32			
